# Complete Genome Sequences of the Two Strains Methylorubrum extorquens NBC_00036 and NBC_00404

**DOI:** 10.1128/mra.00115-23

**Published:** 2023-06-20

**Authors:** David Faurdal, Tue S. Jørgensen, Maria Alvarez-Arevalo, Anna-Sophie Mourched, Eva B. Sterndorff, Pep Charusanti, Tilmann Weber

**Affiliations:** a The Novo Nordisk Foundation Center for Biosustainability, Technical University of Denmark, Kongens Lyngby, Denmark; The University of Arizona

## Abstract

Here, we report the complete genome sequences of Methylorubrum extorquens NBC_00036 and *Methylorubrum extorquens* NBC_00404. The genomes were sequenced using the Oxford Nanopore Technologies MinION and Illumina NovaSeq systems. Both genomes are circular, with sizes of 5,661,342 bp and 5,869,086 bp, respectively.

## ANNOUNCEMENT

Soil-derived bacteria have served as a source of many important secondary metabolites. Yet, methylotrophs, common in soil, have not been widely studied for their biosynthetic gene clusters (BGCs) (1). Here, we present the complete genome sequences and biosynthetic potential of two strains of Methylorubrum extorquens, NBC_00036 and NBC_00404.

In 2016, *Methylorubrum extorquens* NBC_00036 and NBC_00404 were isolated from Danish and Spanish soil on ISP-2 medium (2) by mixing 1 g of soil with 3 mL of 6% yeast extract, diluting this mixture 1:49 in sterile water and spreading the dilution onto International *Streptomyces* Project 2 (ISP-2) medium. Single colonies were then picked and used to make glycerol stocks. The strains were inoculated from the glycerol stock directly into liquid ISP-2 and grown at 30°C and 140 rpm for genomic DNA purification using a modified Qiagen Genomic-tip 20/G kit protocol, as described in (3).

Libraries were prepared for Nanopore sequencing using the rapid barcoding 96 kit (SQK-RBK110.96; Oxford Nanopore Technologies, Inc.) and then sequenced on an R9.4.1 flow cell using a MinION device. Barcode 23 was used for NBC_00036, and barcodes 46 and 87 were used for NBC_00404.

The data were basecalled and demultiplexed using Guppy (5.0.17 + 99baa5b, client-server API V7.0.0) applying the high-accuracy model, excluding reads smaller than 1 kb. This yielded 15,270 raw reads for NBC_00036, with an *N*_50_ value of 26,081 bp and a coverage of 34×, and 118,436 raw reads for NBC_00404, with an *N*_50_ value of 11,876 bp and a coverage of 163×.

Next-generation sequencing (NGS) DNA prep set (Novogene) libraries with size selection from 250 to 400 bp were used to generate Illumina NovaSeq data. This yielded 6,310,138 and 7,033,369 2 × 150-nucleotide (nt) read pairs for NBC_00036 and NBC_00404, providing coverages of 324× and 374×, respectively (as reported by Polypolish). The reads were trimmed using Trim Galore with Cutadapt V2.10 to a minimum length of 100 bp with a quality of at least 20 (4).

The Nanopore reads were assembled *de novo* using Flye V2.9 (5), applying --nano-raw and 5 iterations of polishing. The assemblies were then polished with the Illumina reads using Polypolish version 0.5 (6), leading to 1,744 and 6,044 changed positions from NBC_00036 and NBC_00404.

Finally, the assemblies were polished using POLCA (7) (part of MaSuRCA V4.0.5). BUSCO V5.1.2 (8) was used to check the quality of assemblies with the alphaproteobacteria_odb10 data set (8). The strains were identified as members of Methylobacterium extorquens (called *Methylorubrum extorquens* at NCBI) using GTDB-tk V1.7.0 (9), with the closest match being the genome found under GenBank accession number GCF_900234795.1 (ANI, 96.88% and 96.65% for NBC_00036 and NBC_00404, respectively). The genomes were annotated using PGAP V6.3 (10). Finally, the BGCs encoded by the strains were predicted using antiSMASH V6.2 (11), with ClusterBlast, KnownClusterBlast, and SubClusterBlast applied.

The relevant genome statistics, attributes, and predicted BGCs of the two strains are provided in Table 1. The two strains have an average nucleotide identity of 99.33%, determined using FastANI (12). This is also reflected in the fact that the predicted BGCs are almost identical, except for the RiPP-like factor encoded by NBC_00036.

**TABLE 1:** Attributes of *Methylorubrum extorquens* NBC_00036 and NBC_00404

Attribute	Data for strain:	
	NBC_00036	NBC_00404
Mean Nanopore Coverage (×)	34	163
Size (bp)	5,661,342	5,869,086
GC content (%)	68.37	68.29
No. of coding sequences	5,197	5,489
No. of rRNAs	15	15
No. of antiSMASH-predicted BGCs		
Homo serine lactone clusters	1	1
Nonalpha poly-amino acid-like ε-polylysine clusters	1	1
Nonribosomal peptide synthetase clusters	1	1
Redox-cofactors	1	1
RiPP-like factors	1	0
Siderophore clusters	1	1
Type I polyketide synthases	1	1
Terpene clusters	3	3
No. of BUSCOs		
Complete	431	431
Single-copy complete	428	430
Duplicated complete	3	1
Fragmented	0	0
Missing	1	1
Total	432	432

### Data availability.

The data are available under NCBI BioProject accession number PRJNA884983. The raw reads were deposited in the SRA under BioSample accession numbers SAMN31331780 (NBC_00036) and SAMN31331782 (NBC_00404). The genome sequences can be found under GenBank accession numbers CP110131 and CP110130.
